# Selective conversion of CO_2_ and H_2_ into aromatics

**DOI:** 10.1038/s41467-018-05880-4

**Published:** 2018-08-27

**Authors:** Youming Ni, Zhiyang Chen, Yi Fu, Yong Liu, Wenliang Zhu, Zhongmin Liu

**Affiliations:** 1 National Engineering Laboratory for Methanol to Olefins, Dalian Institute of Chemical Physics, Chinese Academy of Sciences, P. O. Box 110 , 116023 Dalian, People’s Republic of China; 2 Dalian National Laboratory for Clean Energy, Dalian Institute of Chemical Physics, Chinese Academy of Sciences, Dalian, 116023 People’s Republic of China; 3 University of Chinese Academy of Sciences, 100049 Beijing, People’s Republic of China

## Abstract

Transformation of greenhouse gas CO_2_ and renewable H_2_ into fuels and commodity chemicals is recognized as a promising route to store fluctuating renewable energy. Although several C_1_ chemicals, olefins, and gasoline have been successfully synthesized by CO_2_ hydrogenation, selective conversion of CO_2_ and H_2_ into aromatics is still challenging due to the high unsaturation degree and complex structures of aromatics. Here we report a composite catalyst of ZnAlO_*x*_ and H-ZSM-5 which yields high aromatics selectivity (73.9%) with extremely low CH_4_ selectivity (0.4%) among the carbon products without CO. Methanol and dimethyl ether, which are synthesized by hydrogenation of formate species formed on ZnAlO_*x*_ surface, are transmitted to H-ZSM-5 and subsequently converted into olefins and finally aromatics. Furthermore, 58.1% *p*-xylene in xylenes is achieved over the composite catalyst containing Si-H-ZSM-5. ZnAlO_*x*_&H-ZSM-5 suggests a promising application in manufacturing aromatics from CO_2_ and H_2_.

## Abstract (web summary)

Selective conversion of CO_2_ and H_2_ into aromatics remains challenging due to the high unsaturation degree and complex structure of aromatics. Here the authors report a composite catalyst of ZnAlO*x* and H-ZSM-5 which promotes the formation of aromatics with high selectivity while inhibiting CO and CH_4_ formation in CO_2_ hydrogenation reactions.

## Introduction

The utilization of fossil resources such as coal, oil, and natural gas has brought us unprecedented economy and social development in the past two centuries^1^, however, continuously increasing the emissions of greenhouse gas CO_2_ is threatening our living environment. The renewable energy resources (for example, solar, tidal, wind and biomass) can generate abundant power, but low-efficiency and fluctuating nature limits their widespread applications^2^. These problems above could be effectively overcome via CO_2_ hydrogenation to fuels (e.g., gasoline) and commodity chemicals (e.g., methanol, olefins and aromatics) because hydrogen can be acquired from the clean electricity^3–6^.

Considering that CO_2_ (Δ_*f*_G^*o*^ = −396 kJ mol^−1^) is a chemical inert molecular^3^, CO_2_ hydrogenation reactions are generally operated under high pressure and hydrogen content over reductive metal catalysts so as to improve the conversion efficiency. Nowadays, C_1_ chemicals such as methanol (MeOH), dimethyl ether (DME), formic acid (HCOOH), methane (CH_4_), and carbon monoxide (CO) have been selectively synthesized under above conditions^3,4,6,7^. However, production of C_2+_ hydrocarbons such as olefins and liquid fuels from CO_2_ are not easy because of high kinetic barriers for C–C coupling^6,8^. In the earlier studies, combination of reverse-water-gas-shift (RWGS, CO_2_ + H_2_ → CO + H_2_O) and Fischer–Tropsch (FT, CO + H_2_ → C_*n*_H_*m*_) synthesis reactions was considered as a promising method to generate long-chain hydrocarbons from CO_2_^2,9^. Nevertheless, due to the restriction of Anderson–Schulz–Flory (ASF) distribution^10,11^, the selectivity of C_2_–C_4_ and gasoline fraction hydrocarbons does not exceed 58% and 48%^12,13^, respectively. More recently, high selective olefins or gasoline has been achieved from CO_2_ hydrogenation via utilization of oxide/zeolite bifunctional catalysts which have been successfully applied in syngas-to-olefins (STO) or aromatics (STA) reactions^14–20^. Some typical results are as follows: In−Zr oxide, ZnGa_2_O_4_ or ZnO-ZrO_2_ combined with SAPO-34 zeolites achieved more than 80% C_2_–C_4_ olefins^21–23^, meanwhile, In_2_O_3_ or Na-Fe_3_O_4_ coupled with H-ZSM-5 zeolites reached to approximately 80% gasoline-range hydrocarbons^24,25^. Because metal catalysts or the Brønsted acid sites of the zeolites can catalyze hydrogenation reactions^26,27^, it is challenging to synthesize aromatics with high unsaturation degree and complex structures under conditions of high H_2_ content. Up to now, there are no reports on the highly selective conversion of CO_2_ and H_2_ into aromatics.

Here, we report a composite catalyst made by nano-scaled spinel structural ZnAlO_*x*_ oxide and H-ZSM-5 zeolite (ZnAlO_*x*_&H-ZSM-5), which exhibits 73.9% aromatics selectivity with only 0.4% CH_4_ selectivity among the carbon products without CO in CO_2_ hydrogenation reaction. RWGS reaction is largely suppressed by increasing H_2_/CO_2_ ratio or introducing CO. 58.1% *p*-xylene in xylenes is achieved over the composite catalyst containing Si-H-ZSM-5. Reaction mechanism and the causes of excellent aromatization performance are also explored.

## Results and Discussion

### Catalytic results

**Fig. 1:**
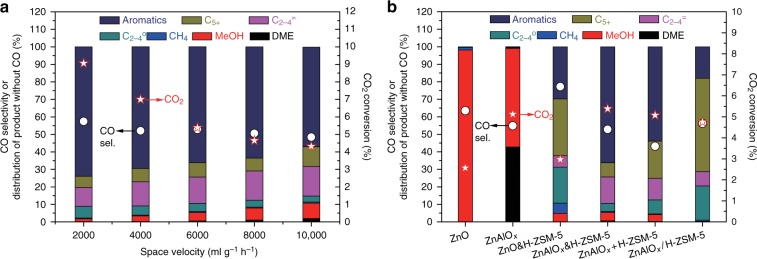
Catalytic performance for CO_2_ hydrogenation. **a** The effect of space velocity over ZnAlO_*x*_&H-ZSM-5. Reaction conditions: 593 K, 3.0 MPa, H_2_/CO_2_/Ar = 3/1/0.2. **b** Comparisons of the CO_2_ conversion and product selectivity over various catalysts. Reaction conditions: Space velocity = 12,000 (for ZnO and ZnAlO_*x*_) or 6000 (for composite catalysts) ml g^−1^ h^−1^, 3.0 MPa, 593 K, H_2_/CO_2_/Ar = 3/1/0.2. Note that the C_5+_ excludes aromatics. C_2-4_^=^ and C_2-4_^o^ refer to C_2_–C_4_ olefins and paraffins, respectively; ZnAlO_*x*_&H-ZSM-5 prepared by grinding; ZnAlO_*x*_ + H-ZSM-5 prepared by granules mixing; ZnAlO_*x*_/H-ZSM-5 denoted as dual-bed catalysts

CO_2_ hydrogenation reactions were conducted over ZnAlO_*x*_&H-ZSM-5 under reaction conditions of H_2_/CO_2_/Ar = 3/1/0.2, pressure 3.0 MPa, and 593 K. The effects of space velocity on CO_2_ conversion and product selectivity are shown in Fig. 1a. It is surprising to find that the selectivity of aromatics among the carbon products without CO reaches as high as 73.9% with 9.1% CO_2_ conversion and 57.4% CO selectivity at space velocity = 2000 ml g^−1^ h^−1^. This aromatics selectivity is much higher than the about 40% obtained over Na-Fe_3_O_4_/H-ZSM-5^25^ or 14.6% acquired over In_2_O_3_/H-ZSM-5^24^, respectively. Besides that, the liquid (or gasoline-range) hydrocarbons including C_5+_ and aromatics (excluding CO) run up to 80.3% along with merely 0.4% CH_4_. As the space velocity substantially rises to 10,000 ml g^−1^ h^−1^, the aromatics selectivity only slightly decreases to 56.9%. This result is quite different from STA reactions, which were often operated at the GHSV <1500 ml g^−1^ h^−1^ so as to peruse high aromatics selectivity^19,20^. It also can be seen from Fig. 1a that MeOH, DME and C_2-4_ olefins are progressively climbing with the space velocity growing, which suggests that these byproducts might be acted as the intermediates for aromatization. The CO_2_ hydrogenation behaviors of various catalysts are compared in Fig.1b. ZnO exhibits 98.2% MeOH selectivity (excluding CO) with 2.6% CO_2_ conversion and 63.5% CO selectivity. After mixed with H-ZSM-5, only 29.7% aromatics selectivity was achieved. Compared to ZnO, ZnAlO_*x*_ shows a higher CO_2_ conversion (5.1%) but lower CO selectivity (54.9%). The DME and MeOH selectivity is up to 42.8% and 56.2% over ZnAlO_*x*_, respectively. It implies that AlO_*x*_ component is acted as the active site for MeOH dehydration. In contrast with the composite catalyst ZnO&H-ZSM-5, the composite catalyst ZnAlO_*x*_&H-ZSM-5 shows a much better aromatization performance, over which 66.2% aromatic selectivity is obtained under the same reaction conditions. As shown in Fig. 1b, although the CO_2_ conversion and liquid hydrocarbons selectivity are approximate, the aromatics selectivity for ZnAlO_*x*_&H-ZSM-5 prepared by two components grinding in an agate mortar is 12.4% higher than ZnAlO_*x*_ + H-ZSM-5 prepared by the granules of two components mixing. It is apparent that the proximity of oxides and zeolites for the former catalyst is better than the latter one. It means that the closer proximity favors the selective generation of aromatics. As a comparison, the dual-bed configuration catalyst ZnAlO_*x*_/H-ZSM-5 with H-ZSM-5 downstream from the ZnAlO_*x*_ provided the poorest behavior with 17.9% aromatics selectivity. The effect of reaction temperature on the CO_2_ hydrogenation over ZnAlO_*x*_&H-ZSM-5 is investigated. Although raising temperature can increase the CO_2_ conversion, the ability of aromatization is considerably weakened (Supplementary Fig. 1). The optimized weight ratio of oxide/zeolite is 1:1 (Supplementary Fig. 2). We also studied the CO_2_ hydrogenation over the mixture of conventional methanol synthesis catalyst CuZnAlO_*x*_ and H-ZSM-5. As shown in Supplementary Fig. 3, the RWGS reaction is predominant, which leads to more than 95% CO selectivity. According to the conditions for CO_2_ hydrogenation, CO hydrogenation over ZnAlO_*x*_&H-ZSM-5 was investigated at H_2_/CO/Ar = 3/1/0.2, space velocity = 6000 ml g^−1^ h^−1^, pressure 3.0 MPa, and 593 K. As depicted in Supplementary Fig. 4, the aromatics selectivity (excluding CO_2_) is only 12.8% with 4.7% CO conversion. It implies that the excellent aromatization behavior of ZnAlO_*x*_&H-ZSM-5 in CO_2_ hydrogenation is not related to self-promotion mechanism of CO reported by Cheng et al.^19^.

### Catalytic stability

**Fig. 2:**
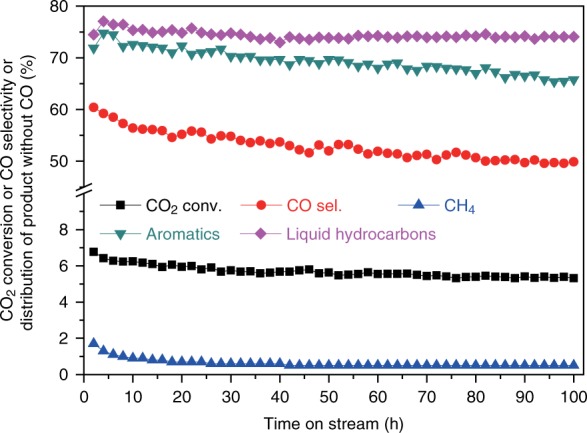
The stability test for CO_2_ hydrogenation over ZnAlO_*x*_&H-ZSM-5. Reaction conditions: Space velocity = 6000 ml g^−1^ h^−1^, 3.0 MPa, 593 K, H_2_/CO_2_/Ar = 3/1/0.2. Note that the liquid hydrocarbons include aromatics

The catalytic stability of ZnAlO_*x*_&H-ZSM-5 in CO_2_ hydrogenation reaction was studied. It is apparent from Fig. 2 that the composite catalyst delivers a good stability in a 100 h test. The CO_2_ conversion, liquid hydrocarbons, and CH_4_ selectivity among the carbon products without CO levels off at around 5.4, 74.0, and 0.5% after 50 h on stream. Although the aromatics selectivity is slightly decreased from 72.0% in the initial state to 66.0% after 100 h on stream, the formation rate of aromatics can keep about 15 mg g^−1^ h^−1^ because the CO selectivity progressively declines.

### Selectivity optimization

**Fig. 3:**
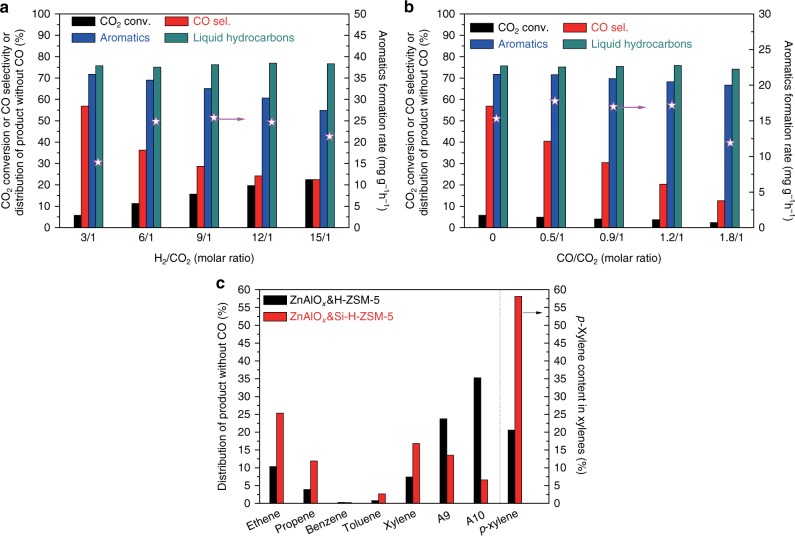
The results of selectivity optimization for CO_2_ hydrogenation. **a** The effect of H_2_/CO_2_ ratio over ZnAlO_*x*_&H-ZSM-5. Reaction conditions: Space velocity = 6000 ml g^−1^ h^−1^, 3.0 MPa, 593 K. **b** The effect of CO introduction over ZnAlO_*x*_&H-ZSM-5. Reaction conditions: catalyst weight = 667 mg, 3.0 MPa, 593 K, inlet gas containing 66.7 ml min^−1^ mixed gas (H_2_/CO_2_/Ar = 3/1/0.2) and 0–26.6 ml min^−1^ CO. **c** Comparisons of catalytic behaviors over ZnAlO_*x*_&H-ZSM-5 and ZnAlO_*x*_&Si-H-ZSM-5. Reaction conditions: Space velocity = 6000 ml g^−1^ h^−1^, 3.0 MPa, 593 K, H_2_/CO_2_/Ar = 3/1/0.2. A9 and A10 are representative of aromatics containing 9 and 10 carbon atoms, respectively

In order to improve the efficiency for CO_2_ hydrogenation to valuable hydrocarbons such as aromatics, light olefins and gasoline, the RWGS reaction should be suppressed as much as possible. It is very interesting to find from Fig. 3a that as the H_2_/CO_2_ ratio rises from 3/1 to 9/1, the CO_2_ conversion is dramatically increased from 5.8 to 15.7% with the CO selectivity sharply decreasing from 56.9 to 28.7%. It means that the generated CO can be further converted by hydrogenation in the high H_2_/CO_2_ ratio. Although raising H_2_/CO_2_ ratio can slightly decrease aromatics selectivity, their formation rate keeps growing. Further increasing H_2_/CO_2_ ratio to 15/1 leads to a decline in aromatization rate. Moreover, the liquid hydrocarbons are not sensitive to H_2_/CO_2_ ratio. As shown in Fig. 3b, another effective method to weaken RWGS reaction is adding CO into the mixture gas of CO_2_ and H_2_. The CO selectivity is nearly monotonically decreasing with CO/CO_2_ ratio increasing and as low as 12.6% at CO/CO_2_ ratio = 1.8/1, nevertheless, the aromatics or liquid hydrocarbons selectivity is slightly influenced after CO introduction. As known, RWGS is a typical equilibrium reaction^4^. Therefore, introduction product CO does not benefit the formation of itself. These results above are vital to applications. On the one hand, the source of feed gas is more extensive because its composition can be adjusted in a wide range; on the other hand, the product CO can be recycled and accumulated so as to practically depress the RWGS reaction. As presented in Fig. 3c, aromatics with 9 to 10 carbons are predominant over ZnAlO_*x*_&H-ZSM-5. We tried to optimize the distributions of aromatics by using Si-H-ZSM-5 made by tetraethoxysilane (TEOS) modification. Compared to ZnAlO_*x*_&H-ZSM-5, ZnAlO_*x*_&Si-H-ZSM-5 obviously produces more toluene (2.7%) and xylenes (16.8%) (Fig. 3c). Moreover, 58.1% *p*-xylene in xylenes, 25.3% ethylene and 11.9% propylene (excluding CO) along with little C_1-3_ alkanes are obtained over ZnAlO_*x*_&Si-H-ZSM-5 (Fig. 3c and Supplementary Fig. 5). The catalytic behavior for ZnAlO_*x*_&Si-H-ZSM-5 would be interesting to industry, because ethylene, propylene and *p*-xylene are the most important commodity hydrocarbon chemicals.

### Structural characterization

**Fig. 4:**
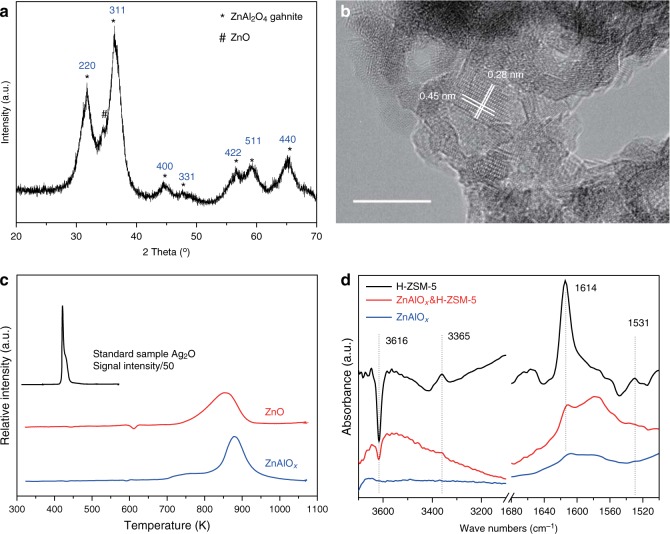
Structural characterization of various catalysts. **a** X-ray diffraction patterns (XRD) of ZnAlO_*x*_. **b** High-resolution transmission electron microscopy (TEM) image of ZnAlO_*x*_. Scale bar: 10 nm. **c** H_2_-TPR profiles. The relative intensity was normalized by weight. The relative intensity of the standard sample Ag_2_O was divided by 50. **d** FTIR subtraction spectra relative to adsorption of DTBPy

Figure 4a shows the X-ray diffraction (XRD) patterns of ZnAlO_*x*_ oxide. The reflection peaks of ZnAlO_*x*_ can be approximately assigned to cubic ZnAl_2_O_4_ gahnite^28^ (JCPDS 05-0669, Supplementary Fig. 6). The reflection peaks of ZnO (Supplementary Fig. 7) are hardly detectable for ZnAlO_*x*_. The Field-emission scanning electron microscopy (SEM) image (Supplementary Fig. 8) shows that the morphology of ZnAlO_*x*_ is porous and made by small particles. The BET surface area of ZnAlO_*x*_ (Supplementary Table 1) is up to 151.0 m^2^ g^−1^, which is much higher than ZnO (11.9 m^2^ g^−1^). High-resolution transmission electron microscopy (TEM) image in Fig. 4b gives us the fact that the ZnAlO_*x*_ sample consists of a lot of tiny nanoparticles with the sizes of <10 nm. Moreover, the lattice spaces of 0.28 and 0.45 nm which respectively correspond to the (220) and (111) planes of cubic ZnAl_2_O_4_ further confirm the spinel structure of ZnAlO_*x*_^28^. The element distribution analysis (Supplementary Fig. 9) shows that the Zn, Al, and O are uniformly dispersed. As presented in Supplementary Fig. 10, the ZnO sample in this study is consisted of about 100 nm nanoparticles with a clear lattice space. The H-ZSM-5 zeolite embraces a typical nano-sized MFI structure (Supplementary Fig. 11). The acidcontent of H-ZSM-5 is 1.3 times as high as Si-H-ZSM-5 (Supplementary Table 2). The temperature-programmed reduction (TPR) results in Fig. 4c and Supplementary Table 3 show that both of ZnAlO_*x*_ and ZnO can be hardly reduced by H_2_ below 700 K and <7% zinc oxides might be reduced to their metal phase above 1000 K. As known, Zn^2+^ cannot be reduced by H_2_ due to the positive free Gibbs energy^29^. However, the reduction may proceed if H_2_ is split into hydrogen atoms or alloy phase with other metals like Cu is formed^30^. Furthermore, the spinel structure of ZnAlO_*x*_ has been proved to be more durable under H_2_ reduction^31^. As a result, we consider that it is not zinc metal but Zn^2+^ in ZnAlO_*x*_ activates CO_2_ hydrogenation reactions in this study. For a long time, the debate about the role of zinc species in industrial Cu/ZnO/Al_2_O_3_ catalysts for methanol synthesis has always existed. ZnO in Cu/ZnO/Al_2_O_3_ was not reckoned as catalytic centers but structural promoter or others^32,33^. However, our results prove that in the absence of Cu species, ZnAlO_*x*_ can also convert CO_2_ into MeOH and derived DME. The FT-IR spectra of catalysts after 2,6-di-tert-butyl-pyridine absorption (DTBPy-FTIR) are exhibited in Fig. 4d. DTBPy-FTIR was usually utilized to investigate the external surface Brønsted acid sites because the kinetic diameter (10.5 Angstrom) of DTBPy is larger than the pore opening (5.5 Angstrom) of H-ZSM-5. The characteristic bands at 3365, 1614 and 1531 cm^−1^ are attributed to the DTBPy adsorbed on the external surface Brønsted acid sites of H-ZSM-5 zeolite. The negative band at 3616 cm^−1^ is assigned to the decrease of bridging hydroxyls on the external surface^34^. Surprisingly, these typical bands almost disappeared for the composite catalyst ZnAlO_*x*_&H-ZSM-5. It indicates that external Brønsted acid of H-ZSM-5 can be shielded by ZnAlO_*x*_ after mixing, grinding and pressing under high pressure. The external Brønsted acid has no ability to aromatization but can promote the hydrogenation of unsaturated hydrocarbons to paraffins^15,26,27^, which is detrimental to the aromatics selectivity. The aromatics or C_2-4_ olefins selectivity follows the order ZnAlO_*x*_&H-ZSM-5˃   ZnAlO_*x*_ + H-ZSM-5>ZnAlO_*x*_/H-ZSM-5 (Fig. 1b), whereas the order of C_2-4_ paraffins selectivity completely reverses, which verifies this point of view.

### *Operando* DRIFT study

**Fig. 5:**
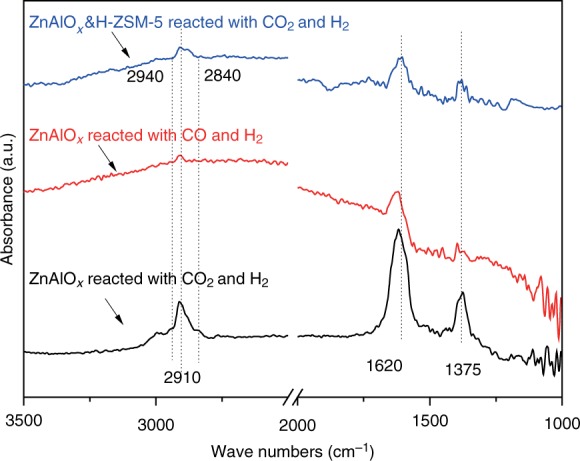
*Operando* DRIFT spectra over various catalysts. Conditions: 593 K, 0.1 MPa. CO_2_&H_2_ refers to the mixed gas of H_2_/CO_2_/Ar = 3/1/0.2; CO&H_2_ refers to the mixed gas of H_2_/CO/Ar = 3/1/0.2

The *operando* diffuse reflectance infrared Fourier transform spectroscopy (DRIFTS) of CO_2_ hydrogenation over ZnAlO_*x*_ at 593 K and 0.1 MPa are explored. It is obvious from Supplementary Fig. 12 that absorbed surface formate species were first formed, then, absorbed surface methoxy species were generated by hydrogenation of formate species. As presented in Fig. 5, compared to DRIFTS of CO_2_ hydrogenation over ZnAlO_*x*_, the bands for surface formate (1620, 1375 and 2910 cm^−1^) and methoxy (2940 and 2840 cm^−1^) species^35,36^ over ZnAlO_*x*_&H-ZSM-5 are sharply weakened, which suggests that these active surface species over ZnAlO_*x*_ were converted by H-ZSM-5. Combined the catalytic results in Fig. 1a with DRIFTS results discussed above, we propose a mechanism for CO_2_ hydrogenation to aromatics over ZnAlO_*x*_&H-ZSM-5. First, surface formate species on ZnAlO_*x*_ was hydrogenated to form surface methoxy species; then, intermediates including MeOH and DME produced from the dissociation of methoxy species were spread to H-ZSM-5 to synthesize olefins intermediates; finally, transformation of olefins to aromatics consecutively take place in the micropores of H-ZSM-5. Furthermore, compared to CO_2_ hydrogenation over ZnAlO_*x*_, CO hydrogenation generates much less surface formate species. That is to say, more active metal species sites of ZnAlO_*x*_ are utilized to activate H_2_ during CO hydrogenation, so that higher C_2-4_ paraffins but lower aromatics are obtained in Supplementary Figure 5 because hydrogenation reactions of intermediates like olefins are enhanced. From Supplementary Fig. 13, we believe that CO_2_ hydrogenation cannot proceed over H-ZSM-5. Some evidences were put forward by Jiao et al.^14,17,20^ to support the ketene intermediate mechanism for STO or STA reactions over oxides/zeolites composite catalysts, however, we consider that this mechanism does not work in CO_2_ hydrogenation over ZnAlO_*x*_&H-ZSM-5 due to very low concentration of CO in the reaction process.

In summary, composite catalyst of ZnAlO_*x*_ with a nano-scaled spinel structure and H-ZSM-5 exhibits an excellent performance for CO_2_ hydrogenation to aromatics. The selectivity of aromatics (excluding CO) reaches as high as 73.9% with 9.1% CO_2_ conversion and 0.4% CH_4_ selectivity. Increasing H_2_/CO_2_ ratio or introducing CO can effectively suppress RWGS reaction without obviously weakening aromatization. The composite catalyst containing Si-H-ZSM-5 presents higher *p*-xylene, ethylene and propylene selectivity. It is not zinc metal but Zn^2+^ in ZnAlO_*x*_ activates CO_2_ hydrogenation. The shield of the external Brønsted acid of H-ZSM-5 by ZnAlO_*x*_ is beneficial to aromatization. MeOH, DME and olefins are acted as reaction intermediates. CO_2_ hydrogenation over ZnAlO_*x*_&H-ZSM-5 shows much higher aromatics selectivity than CO hydrogenation because the former reaction generates more formate species on ZnAlO_*x*_ surface so that less metal species sites are left for hydrogenation of unsaturated intermediates like olefins. ZnAlO_*x*_&H-ZSM-5 suggests a promising application in manufacturing aromatics from CO_2_ and H_2_.

## Methods

### Catalyst preparation

ZnAlO_*x*_ oxide was prepared by a conventional co-precipitation method. Typically, solution A was made by 59.5 g Zn(NO_3_)_2_ 6H_2_O and 75.0 g Al(NO_3_)_3_ 9H_2_O dissolved in 150 ml deionized water and solution B was prepared by 23.5 g (NH_4_)_2_CO_3_ dissolved in 150 ml deionized water. Solutions A and B were dividedly added into one beaker by two peristaltic pumps with constant pH of 7.1–7.3 at 343 K under continuous stirring for 0.5 h, then followed by aging for 3 h at the same temperature. After filtered and washed by 200 ml deionized water for three times, the obtained mixture was dried at 373 K overnight and then calcined at 773 K for 4 h.

Commercial Na-ZSM-5 zeolite was supplied from Zhongke New Catalytic Technology Company, China. Na-ZSM-5 zeolites were transformed into NH_4_-ZSM-5 by exchanging 50 g Na-ZSM-5 with 0.5 l NH_4_NO_3_ (1 mol l^−1^) aqueous solution at 353 K for 2 h, followed by filtration and washing with deionized water. After repeating the exchanging process for three times, the resultant samples were dried overnight at 373 K, followed by calcination at 823 K for another 4 h in air to obtain the H-ZSM-5 zeolite.

Si-H-ZSM-5 zeolite was prepared by tetraethoxysilane (TEOS) modification. In brief, an organic solution was firstly made by 11.5 g TEOS mixed with 5 ml cyclohexane; then 10.0 g H-ZSM-5 was added into this solution, impregnated for 24 h at room temperature; after that, organic solvent was removed by evaporation at 353 K and the resultant powder was dried overnight at 373 K, followed by calcination at 823 K for another 4 h in air. After repeating the procedure above for twice, the Si-H-ZSM-5 was obtained.

The composite catalysts were typically prepared by physical mixing. Unless specially stated, the weight ratio of oxides and zeolites was 1:1. For preparation of the composite catalyst ZnAlO_*x*_&H-ZSM-5, ZnAlO_*x*_ oxide and H-ZSM-5 zeolite were grinded in an agate mortar for 4 min, pressed under 40 MPa and granulated into the required size in the range of 0.4–0.8 mm.

### Catalytic tests

Catalytic reaction experiments in Fig.1 and Supplementary Figure 2 and 3 were performed in a 16-channel continuous flow fixed-bed stainless steel reactor (from Yashentech Corporation, Shanghai, China) with an 8.3 mm inner diameter for each channel. The inlet gas flow and composition along with the reaction temperature and pressure for each channel were identical. All the reaction products were kept in the gas phase and analyzed online by two gas chromatographs (Aglient 7890A) equipped with a HP-PLOT/Q capillary column connected to a flame ionization detector (FID) and a TDX-1 column (produced from DICP) connected to a thermal conductivity detector (TCD). CH_4_ was taken as a reference bridge between FID and TCD. Ar was used as an inner standard. The CO_2_ conversion and the CO selectivity, hydrocarbons (C_*n*_H_*m*_), MeOH and DME selectivity among the carbon products without CO (including C_*n*_H_*m*_, MeOH and DME) were calculated with the followed equations.

1 $${\mathrm{CO}}_2\,{\mathrm{conversion}} = \left( {{\mathrm{CO}}_{2{\mathrm {in}}} - {\mathrm{CO}}_{2{\mathrm {out}}}} \right)/\left( {{\mathrm{CO}}_{2{\mathrm {in}}}} \right) \times 100\%$$

CO_2in_: moles of CO_2_ at the inlet; CO_2out_: moles of CO_2_ at the outlet;

2 $${\mathrm{CO}}\,{\mathrm{selectivity}} = {\mathrm{CO}}_{{\mathrm {out}}}/\left( {{\mathrm{CO}}_{2{\mathrm {in}}} - {\mathrm{CO}}_{2{\mathrm {out}}}} \right) \times 100\%$$

CO_out_: moles of CO at the outlet;

$$\begin{array}{ccccc}\\ & {\mathrm{C}}_n{\mathrm{H}}_m\,{\mathrm{selectivity}} = {\mathrm{N}}_{{\mathrm{C}}{n}{\mathrm{H}}{m}}/\left( {{\mathrm{total}}\,{\mathrm{carbon}}\,{\mathrm{atoms}}\,{\mathrm{of}}} \right.\\ \\ & \left. {{\mathrm{products}}\,{\mathrm{detected}}\,{\mathrm{by}}\,{\mathrm{FID}}} \right) \times 100\% \\ \\ & {\mathrm{MeOH}}\,{\mathrm{selectivity}} = {\mathrm{N}}_{\mathrm {MeOH}}/\left( {{\mathrm{total}}\,{\mathrm{carbon}}\,{\mathrm{atoms}}\,{\mathrm{of}}} \right. \\ \\ & \left. {{\mathrm{products}}\,{\mathrm{detected}}\,{\mathrm{by}}\,{\mathrm{FID}}} \right) \times 100\%\\ \\ & {\mathrm{DME}}\,{\mathrm{selectivity}} = {\mathrm{N}}_{\mathrm {DME}}/\left( {{\mathrm{total}}\,{\mathrm{carbon}}\,{\mathrm{atoms}}\,{\mathrm{of}}\,} \right. \\ \\ & \left. {{\mathrm{products}}\,{\mathrm{detected}}\,{\mathrm{by}}\,{\mathrm{FID}}} \right) \times 100\%\end{array}$$

N_C*n*__H*m*_: carbon atoms number of C_*n*_H_*m*_; N_Me6OH_: carbon atoms number of MeOH; N_DME_: carbon atoms number of DME.

Other catalytic reaction experiments were performed in a typical fixed-bed stainless steel reactor with 8 mm inner diameter. All products were analyzed online by two tandem gas chromatographs. One is Agilent 7890A equipped with a HP-PLOT/Q capillary column connected to FID and a TDX-1 column connected to TCD. The other one is Agilent 6890N equipped with a HP-FFAP capillary column connected to FID and a Porapak Q column connected to TCD. The detailed analysis for aromatics such as ethylbenzene, *p*-xylene, *m*-xylene and *o*-xylene is dependent on HP-FFAP capillary column.

## Electronic supplementary material

Supplementary Information

## Data Availability

The data supporting the findings of this study are available within the article and its Supplementary Information files. All other relevant source data are available from the corresponding author upon reasonable request.
