# Supplementary material for: Selective conversion of CO2 and H2 into aromatics
Source: Nat Commun. 2018 Aug 27;9:3457. doi: 10.1038/s41467-018-05880-4 (PMC6110867; doi:10.1038/s41467-018-05880-4)
Supplement: Supplementary file 1 — Supplementary Information [file 41467_2018_5880_MOESM1_ESM.pdf]

Selective conversion of CO_2 and H_2 into aromatics

Ni et al.

Supporting information

Supplementary Methods

Catalysts characterization.

The XRD results were recorded on a PANalytical X'Pert PRO X-ray diffractometer with Cu Kα radiation. The chemical composition was determined on a Philips Magix-601 X-ray fluorescence (XRF) spectrometer. SEM measurements were carried out on an SU8020 scanning electron microscopy. TEM images and element mappings were obtained on a JEM-2100 microscope at 200 kV. The BET surface areas were detected by N_2 adsorption at 77 K on a Micromeritics ASAP 2020 system and calculated by the standard multi-point method. H_2-TPR profiles were tested by a Micromeritics AutoChem II 2920 equipped with a TCD detector in a 30 ml min^{-1} H_2 /Ar (H_2/Ar=10/90) mixture with a ramping rate of 10 K min^{-1}. NH_3-TPD results were also tested by a Micromeritics AutoChem II 2920 equipped with a TCD detector. The adsorption of NH_3 was performed at 373 K in a 30 ml min^{-1} NH_3/He (NH_3/He=5/95) mixture for 1 h, and desorption was performed in He flow by raising the temperature to 873 K at a rate of 10 K min^{-1}. *Operando* DRIFTS were detected on a Bruker Tensor 27 instrument with a MCT detector. The catalyst powder was pressed into a diffuse reflectance infrared cell with ZnSe window. First, the catalyst was treated by 30 ml min^{-1} H_2/Ar (H_2/Ar=3/7) mixture at 593 K and 0.1 MPa for 30 min and the background spectrum was collected under the same conditions. Subsequently, 30 ml min^{-1} mixed gas (H_2/CO_2/Ar= 3/1/0.2 or H_2/ CO/ Ar = 3/1/0.2) was introduced at 593 K and 0.1 MPa and the *operando* DRIFT spectra obtained by collecting 32 scans at 4 cm^{-1} resolution were recorded. FTIR spectra after 2, 6-di-tert-butyl-pyridine (DTBPy) adsorption were obtained on a Bruker Tensor 27 instrument with a resolution of 4 cm^{-1}. Before each measurement, samples were pressed into a self-supporting wafer and evacuated in an IR cell at 673 K for 1 h, then cooled to 423 K and the background spectra were collected. Adsorption of DTBPy was conducted at 423 K for 15 min to ensure saturated loading and then evacuation was sufficiently carried out before each measurement.

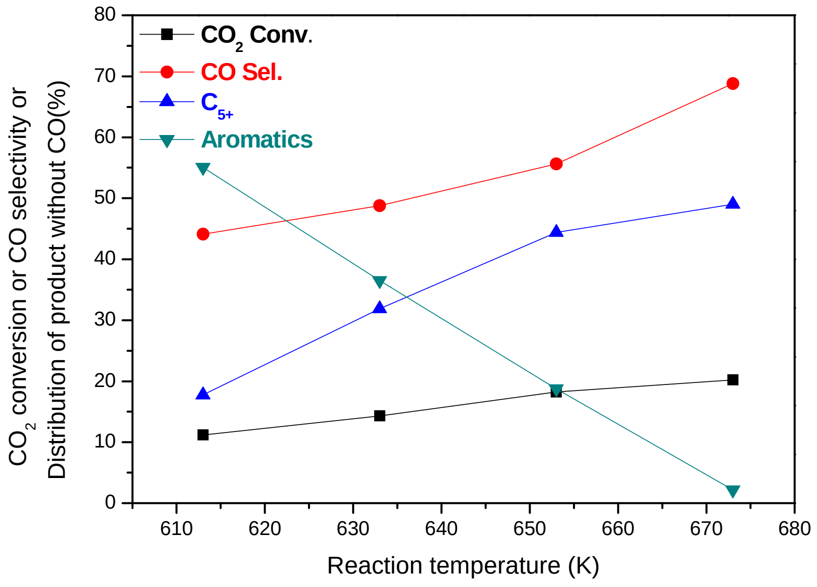

Supplementary Figure 1 | Influence of reaction temperature on the CO_2 hydrogenation over ZnAlO$_x$&H-ZSM-5. Reaction conditions: Space velocity = 6000 ml g^{-1} h^{-1}, 3.0 MPa, $H_2/CO_2/Ar$= 3/1/0.2. Note that the C_{5+} excludes aromatics.

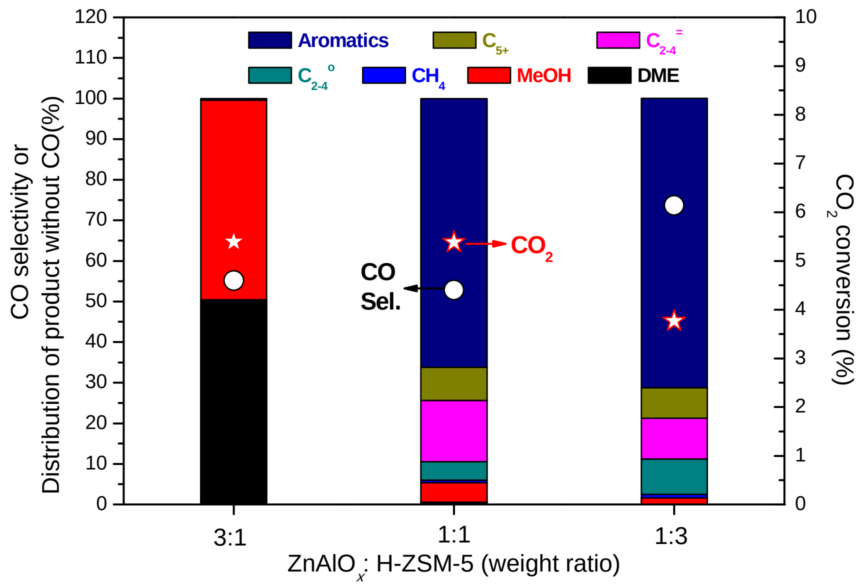

Supplementary Figure 2 | The effect of the weight ratio of Oxides/Zeolites on the CO_2 hydrogenation over $ZnAlO_x$&H-ZSM-5. Reaction conditions: Space velocity = 6000 ml g^{-1} h^{-1}, 3.0 MPa, 593 K, $H_2/CO_2/Ar$= 3/1/0.2. Note that the C_{5+} excludes aromatics. $C_{2-4}^{=}$ and C_{2-4}^{o} refer to C_2-C_4 olefins and paraffins, respectively.

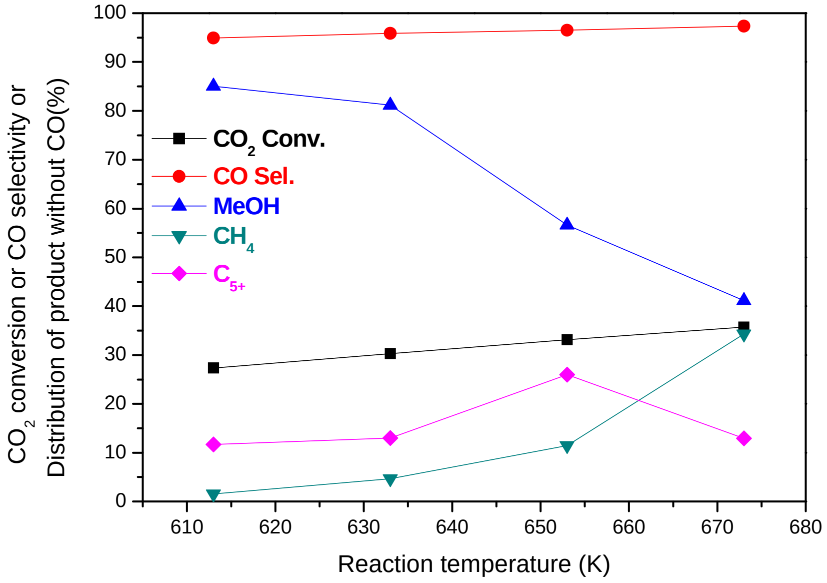

Supplementary Figure 3 | Influence of reaction temperature on the CO_2 conversion over $CuZnAlO_x$&H-ZSM-5. Reaction conditions: Space velocity = 5000 ml g^{-1} h^{-1}, 3.0 MPa, $H_2/CO_2/Ar$= 3/1/0.2, $CuZnAlO_x$:H-ZSM-5 (weight ratio) = 1:1. Note that the C_{5+} excludes aromatics.

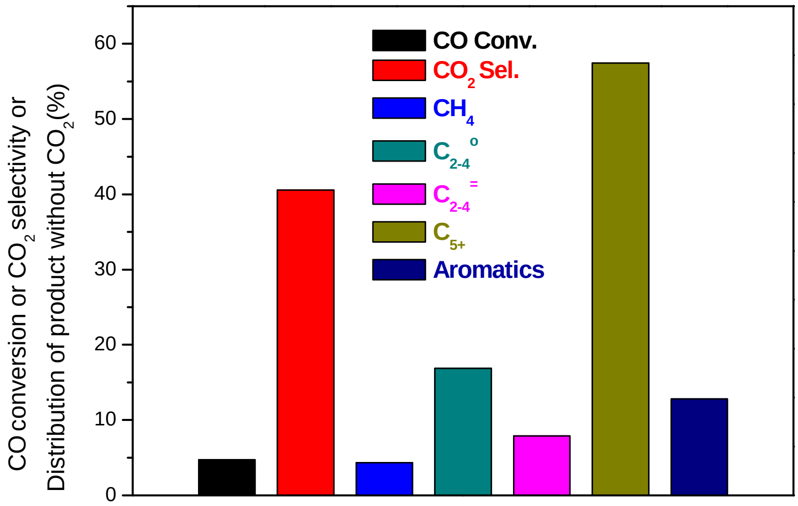

Supplementary Figure 4 | Results of the CO hydrogenation over ZnAlO$_x$&H-ZSM-5. Reaction conditions: Space velocity = 6000 ml g^{-1} h^{-1}, 3.0 MPa, 593 K, H_2/CO/Ar= 3/1/0.2. Note that the C_{5+} excludes aromatics. $C_{2-4}^{=}$ and C_{2-4}^{o} refer to C_2-C_4 olefins and paraffins, respectively.

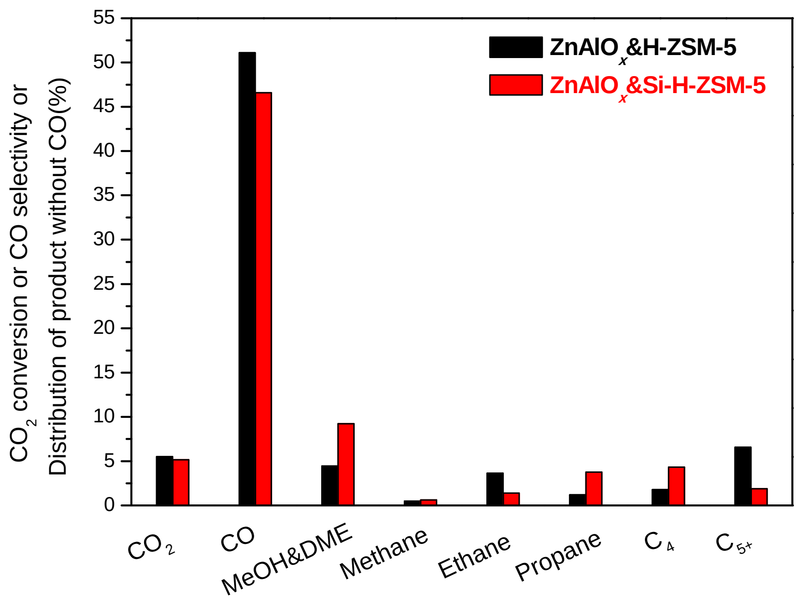

Supplementary Figure 5 | Comparisons of catalytic behaviors over ZnAlO$_x$&H-ZSM-5 and ZnAlO$_x$&Si-H-ZSM-5. Reaction conditions: Space velocity = 6000 ml g^{-1} h^{-1}, 3.0 MPa, 593 K, $H_2/CO_2/Ar$= 3/1/0.2. Note that the C_{5+} excludes aromatics.[1]

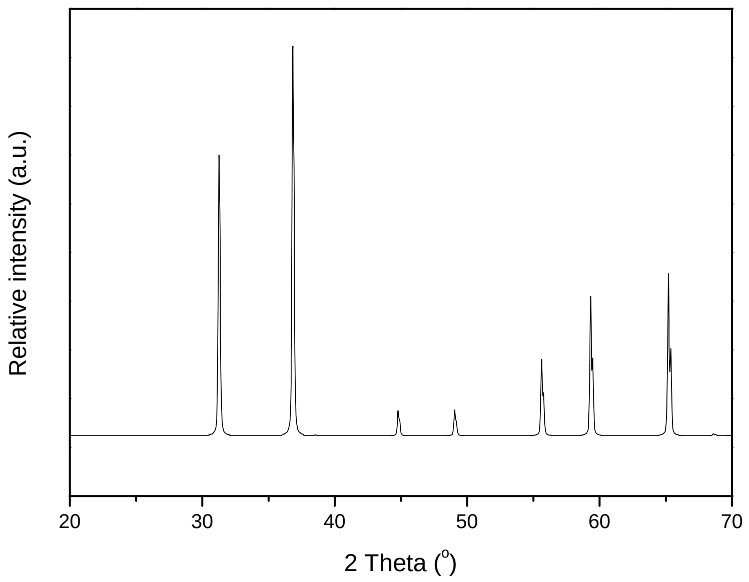

Supplementary Figure 6 | XRD patterns of cubic $ZnAl_2O_4$ gahnite.

(JCPDS 05-0669).

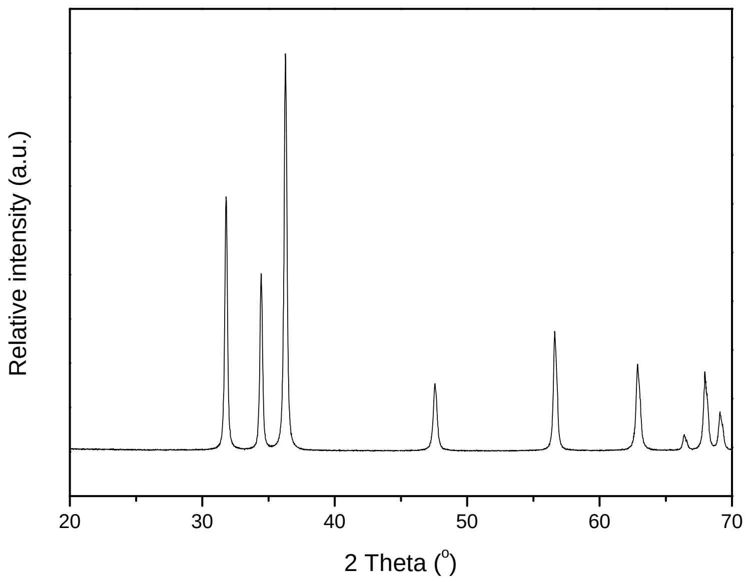

Supplementary Figure 7 | XRD patterns of ZnO.

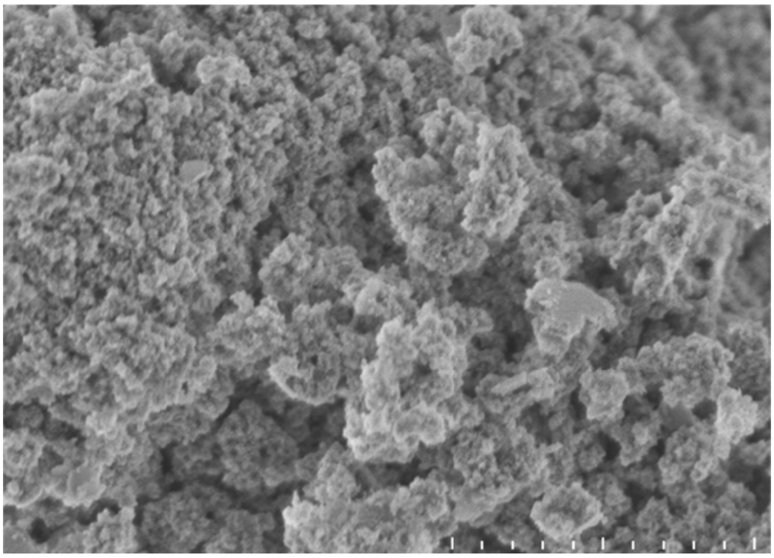

Supplementary Figure 8 | SEM image of $ZnAlO_x$. Scale bar: 1.0 μm.

Supplementary Table 1 | The elemental content and BET surface area for oxides

Oxides name	Zn content [a] (%)	Al content [a] (%)	BET surface area [b] ($m^2\ g^{-1}$)
$ZnAlO_x$	41.5	24.5	151.0
ZnO	80.3	0	11.9

[a] by XRF.

[b] by N_2 physical adsorption-desorption.

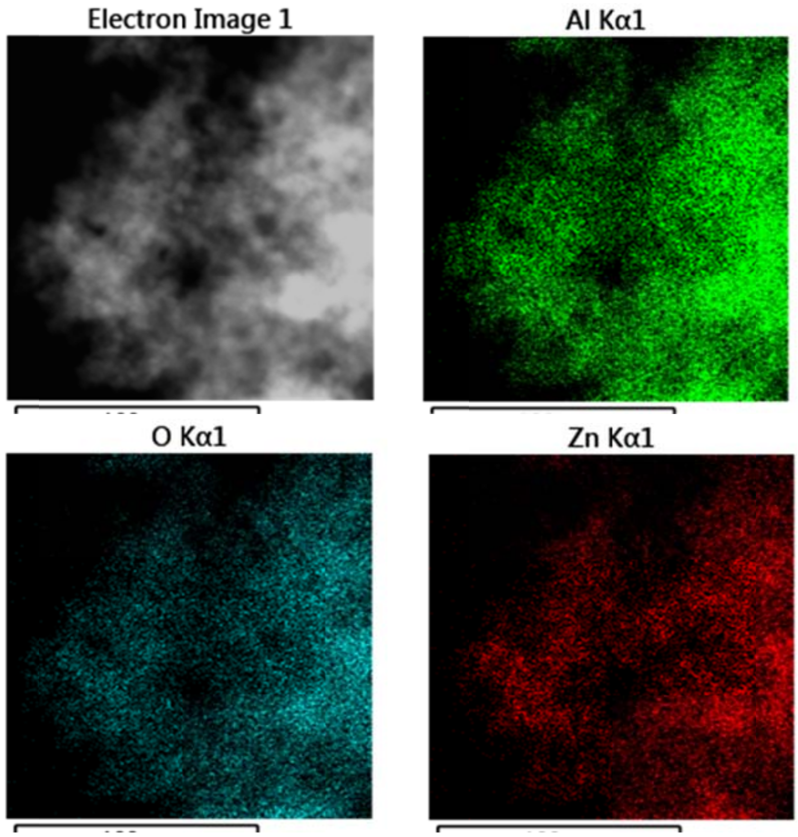

Supplementary Figure 9｜ EDS mapping of Al, O, Zn for $ZnAlO_x$.

Scale bar: 100 nm.

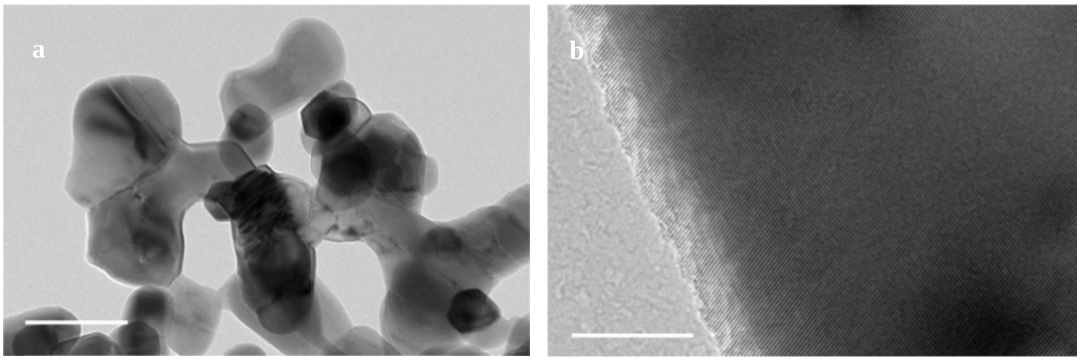

Supplementary Figure 10 | TEM images of ZnO.

(a) Scale bar: 100 nm. (b) Scale bar: 10 nm.

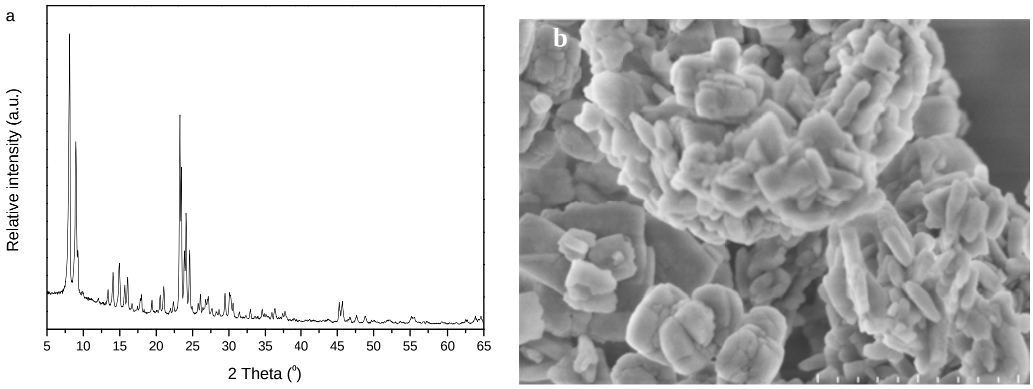

Supplementary Figure 11 | The XRD pattern (a) and SEM image (b) of H-ZSM-5. Scale bar: 1.0 μm.

Supplementary Table 2 | The Si/Al ratio, acid content and BET surface area for zeolites

Zeolite name	Si/Al [a] (%)	Acid content [b] (mmol g^{-1})	BET surface area [c] (m^2 g^{-1})
H-ZSM-5	96.6	0.109	397.0
Si-H-ZSM-5	105.8	0.085	346.8

[a] by XRF.

[b] by NH_3-TPD.

[c] by N_2 physical adsorption-desorption.

Supplementary Table 3 | The results of TPR.

Sample	Weight (mg)	Metal amount (mmol)	Peak temperature (K)	Peak area	H_2 consumption amount (mmol)
Ag_2O	18.2	0.16	412.4	0.47	0.08
ZnO	201.1	2.48	852.3	0.42	0.07
$ZnAlO_x$	200.5	1.28	879.1	0.57	0.10

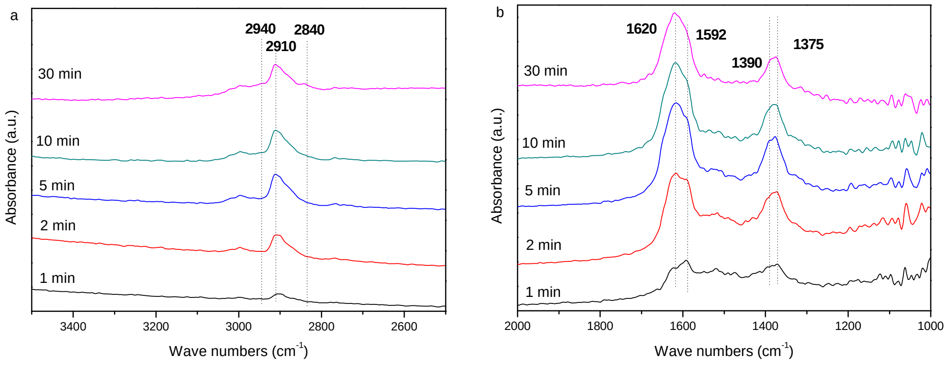

Supplementary Figure 12 | *Operando* DRIFT spectra in CO_2 hydrogenation over $ZnAlO_x$. Conditions: 593 K, 0.1 MPa.

The bands at 1592 and 1620 cm^{-1} are assigned to the asymmetric stretching frequency of the O-C-O bonds of surface bidentate or monodentate formate species (HCOO), respectively. The bands at 1390 and 1375 cm^{-1} are assigned to the symmetric stretching frequency of the O-C-O bonds of surface bidentate or monodentate formate species (HCOO), respectively. The peak at 2910 cm^{-1} is assigned to the stretching vibration *v*(CH) of formate species. The bands at 2940 and 2840 cm^{-1} are attributed to adsorbed methoxy species (CH_3O) [1, 2].

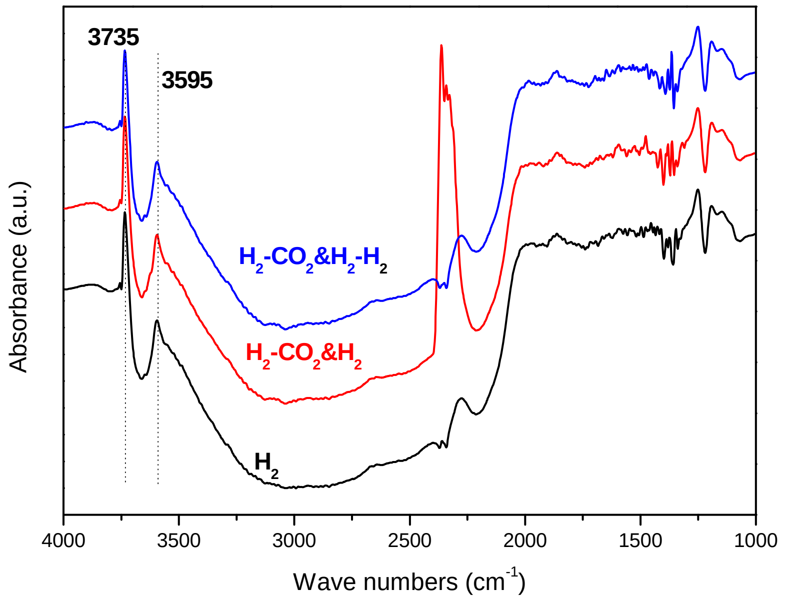

Supplementary Figure 13 | *Operando* DRIFT spectra in CO_2 hydrogenation over H-ZSM-5. Conditions: 593 K, 0.1 MPa. H_2-CO_2&H_2 refers to the reaction with the mixed gas of H_2/CO/Ar= 3/1/0.2 after H_2 treatment. H_2-CO_2&H_2-H_2 refers to H_2 sweeping after H_2-CO_2&H_2 treatment.

The bands at 3735 and 3595 cm^{-1} are assigned to silanol and bridging hydroxyls, respectively.

Supplementary References

1. Le Peltier, F., Chaumette, P., Saussey, J., Bettahar, M. M. & Lavalley, J. C. In situ FT-IR and kinetic study of methanol synthesis from CO_2/H_2 over $ZnAl_2O4$ and Cu–$ZnAl_2O_4$ catalysts. *J. Mol. Catal. A* **132**, 91-100 (1998).
2. Mierczynski, P. *et al.* Cu/$Zn_xAl_yO_z$ supported catalysts (ZnO: Al_2O_3=1, 2, 4) for methanol synthesis. Catal. Today **176**, 21-27 (2011).
